# Buffering of nuclear membrane tension and mechanotransduction by the endoplasmic reticulum revealed by quantitative ALPIN imaging

**DOI:** 10.21203/rs.3.rs-5530637/v1

**Published:** 2024-12-09

**Authors:** Zhouyang Shen, Zaza Gelashvili, Philipp Niethammer

**Affiliations:** 1 Cell Biology Program, Memorial Sloan Kettering Cancer Center, New York, NY 10065, USA; 2 Gerstner Sloan Kettering Graduate School of Biomedical Sciences, New York, NY 10065, USA.; 3 Present address: Bloomberg-Kimmel Institute of Immunotherapy, Department of Oncology, Johns Hopkins University School of Medicine, Baltimore, MD,21287, USA.

**Keywords:** cPLA_2_, ALPS, nuclear membrane tension, mechanotransduction, endoplasmic reticulum, ferroptosis, wound

## Abstract

Nuclear deformation by osmotic shock or necrosis activates the cytosolic phospholipase A2 (cPla_2_) nuclear shape sensing pathway, a key regulator of tissue inflammation and repair. Ca^2+^ and inner nuclear membrane (INM) tension (T_INM_) are believed to mediate nucleoplasmic cPla_2_ activation. The concept implies that T_INM_ persists long enough to stimulate cPla_2_-INM adsorption. However, T_INM_ may instead be rapidly dissipated by the contiguous endoplasmic reticulum (ER), with cPla_2_-INM adsorption reporting rather on changes in Ca^2+^ than T_INM_. The impact of T_INM_ and ER contiguity on nuclear shape sensing and mechanotransduction remains unknown.

To address this gap, we developed the Ca^2+^ insensitive, T_INM_-only biosensor ALPIN (Amphipathic Lipid Packing sensor domain Inside the Nucleus). By quantitative ALPIN imaging, we found that stress-induced ER fragmentation increases T_INM_ and nuclear membrane mechanotransduction in osmotically shocked or ferroptotic cells, permeabilized cell corpses, and at zebrafish wounds *in vivo*. Our findings reveal critical roles for the ER and T_INM_ in nuclear shape sensing and introduce ALPIN as promising tool for studying organelle membrane mechanotransduction in health and disease.

## Introduction

Cells and tissues must distinguish harmless from harmful (i.e., “critical”) deformation to mount adaptive responses that attenuate the stress or increase stress resilience. Adaptation may involve the suppression of muscle contractility, the induction of cytoskeletal-, junction-, or extracellular matrix (ECM)-components during cell differentiation, or acute cell motility that evades the source of mechanical stress ^1–5^.

By causing nuclear shape changes, critical cell body deformation modulates nuclear-cytoplasmic trafficking of transcription factors and the adsorption of peripheral membrane enzymes, such as cPla_2_, to the INM ^6^. Biological membranes strongly resist tension, because increasing the mean distance between phospholipid headgroups in the bilayer exposes their hydrophobic core to water ^7^. Thus, the hydrophobic membrane adsorption of C2- or PLAT- domains, or amphipathic lipid packing sensor (ALPS) motifs is directly correlated with exposure of the hydrophobic core by bilayer stretch, at least on uncurved membranes like those of the INM ^8^.

The membrane binding of cPla_2_’s C2 domain is synergistically regulated by both, T_INM_ and Ca^2+^. T_INM_ enhances cPla_2_-C2’s apparent membrane affinity by ~ three orders of magnitude when Ca^2+^ is constant ^8^. Despite its superior sensitivity, the Ca^2+^ dependent cPla_2_ probe cannot distinguish whether Ca^2+^ or T_INM_ drives its membrane adsorption. Removing Ca^2+^ from the equation has been challenging for a lack of genetically encoded, Ca^2+^ independent stretch sensors. This technical gap along with our incomplete understanding of inter-organelle membrane mechanics raises the possibility that Ca^2+^, not T_INM_, might drive cPla_2_ mediated nuclear shape sensing.

The ER is contiguous with the nuclear membrane (NM). Before INM stretch can expose hydrophobic binding surface, nuclear membrane in-/ and evaginations must be unfolded. So, if a contiguous ER behaves like a large nuclear evagination, T_INM_ may not develop until the ER has completely “unfolded”, i.e., collapsed into the nucleus. To our best knowledge, this has never been observed. Notwithstanding, whether or how the ER affects T_INM_ and nuclear membrane mechanotransduction is fundamentally unknown. Obviously, if the ER were to instantly quench T_INM_ by compensatory membrane flow, T_INM_ cannot serve as nuclear shape signal.

To test the role of T_INM_ as nuclear shape signal, we developed the Ca^2+^ independent, intranuclear lipid packing probe ALPIN and monitored T_INM_ dynamics during nuclear swelling in the presence or absence of a contiguous ER.

## Results

### ER vesiculation correlates with strong cPla_2_ adsorption to the INM

**Figure 1.**
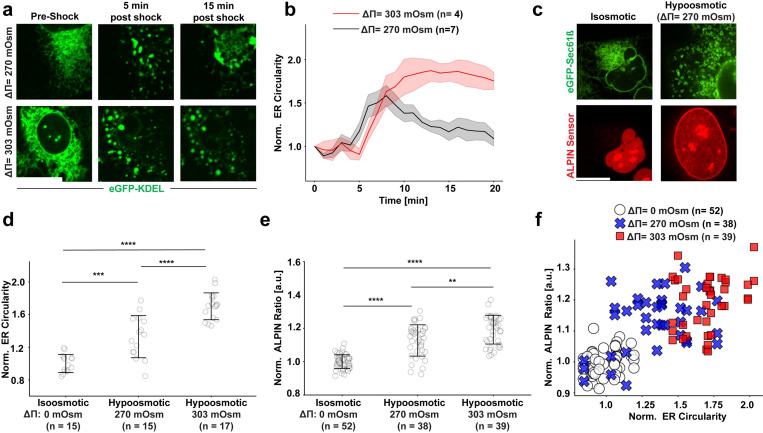
ER vesiculation correlates with strong ALPIN adsorption to the INM. **(a)** Representative images of U2OS cells expressing the ER luminal marker eGFP-KDEL upon mild (Δπ = 270 mOsm) or maximal (Δπ = 303 mOsm) hypoosmotic shock. **(b)** Quantification of ER circularity upon mild (n = 7) or maximal (n = 4) hypoosmotic shock. Shaded areas, standard error of means (SEM). **(c)** Representative images of U2OS cells expressing the ER membrane marker eGFP-Sec 61β and ALPIN, incubated in hypoosmotic (Δπ = 270 mOsm) and isosmotic (Δπ = 0 mOsm) medium (Supplementary Video 2). **(d)** Comparison of ER network circularity of cells in isosmotic (n =15), mild hypoosmotic (n =15) and maximal hypoosmotic (n =17) conditions. Error bars, SD. **(e)** Quantification of ALPIN-NM adsorption in cells incubated in isosmotic (n = 52), mild (n = 38) or extreme hypoosmotic conditions (n = 39). Error bars, standard deviation (SD). **(f)** Scatter plot showing the correlation between ALPIN-NM binding and ER-vesiculation network in cells incubated in mild (n = 38) and extreme hypoosmotic solutions (n = 39) compared to isosmotic medium (n = 52). Note, the plot in (f) is generated from the same raw data plotted in (d) and (e). Scale bars, 20 μm. P values in (d) and (e) are determined by a two-sided Student’s *t*-test assuming unequal variance. For quantification of ER structure in (b) and (d), n represents number of field of views (FOVs) with 1–10 cells each. For measurement of ALPIN-NM binding in (e), n represents number of nuclei. For the correlation plot in (f), n represents number of nuclei. Note that different nuclei in the same FOV are assigned the same ER circularity value (i.e., protein adsorption is assessed per nucleus, whereas ER circularity is assessed per FOV, see Methods).

By imaging the ER-markers eGFP-KDEL (luminal) and eGFP-Sec61b (membrane) along with cPla_2_-INM translocation during osmotic cell-/nuclear swelling, we noticed that strong INM adsorption of cPla_2_ was linked to disrupted ER-NM contiguity. Namely, osmotic shock caused a drastic, partially reversible ER vesiculation (Figure 1a–b, S1, Supplementary Video 1) characterized by a steep decrease of ER network area (Figure S1b, d, g), an inverse increase of ER membrane circularity (Figure S1c, e, h), and strong cPla_2_ adsorption to the INM (Figure S1a, c, f, g, h). A similar overall correlation between ER fragmentation and stable cPla_2_-INM adsorption was observed when nuclei were squeezed (Figure S2a–f), which, at least in our hands, caused irreversible ER-fragmentation. Upon close temporal inspection, we found that cPla_2_-INM adsorption sometimes preceded ER-fragmentation (Figure S2d). ER vesiculation is thus unlikely a prerequisite for T_INM_ and cPla_2_ mediated nuclear shape sensing. Instead, it may block its reversibility through dislodging the ER reservoir from the nuclear membrane. Neither swelling nor squeezing caused nuclear Sytox Orange positivity during the observation window (Figure S3a). Thus, ER fragmentation occurred before cell rupture. Others previously observed reversible ER fragmentation during hypoosmotic shock, Ca^2+^ ionophore treatment, egg fertilization, and neuronal cell death ^9,10,11,12^.

### ALPIN senses T_INM_ independent of Ca^2+^

ER vesiculation presented itself as an experimental opportunity to probe the effect of ER contiguity on T_INM_. As the phenomenon was originally described as a Ca^2+^-dependent process ^10^, we needed to measure T_INM_ independent of Ca^2+^ which is not possible with the cPla_2_ probe. Fortunately, we previously characterized the ALPS domain of ARFGAP1 as potential Ca^2+^ independent membrane tension (T_M_) sensor on giant unilamellar vesicles (GUVs) ^8^. Notably, the domain competes with cPla_2_ for the same tension induced, membrane binding sites ^8^. The ARFGAP1-ALPS domain consists of two 20–40 amino acids long ALPS motifs whose amphiphilic helices insert with their hydrophobic side into the lipid packing defects of highly curved membranes (Figure S4a, b). However, on fairly flat membranes, such as those of the nucleus, ARFGAP1-ALPS binds to stretch induced lipid packing defects just like cPla_2_, albeit with somewhat lower affinity ^8^. The lower membrane affinity is an expected trade-off for obtaining a Ca^2+^ independent T_M_ sensor. Namely, cPla_2_’s extraordinary T_INM_ sensitivity involves the Ca^2+^-dependent amplification of its hydrophobic membrane interactions ^8^.

We targeted fluorescent fusions of the first (ALPS1=ALPIN) or both ALPS motifs (ALPS1–2) of ARFGAP1 to the nucleus with a nuclear localization sequence (3XNLS) (Figure S4c). Both constructs partitioned to the nucleoplasm and nuclear, droplet-like structures (Figure S4d). Nuclear swelling in intact cells was initiated by reducing the osmolarity of the cell culture medium (Figure S4d–g). The dilution of cytoplasmic macromolecules by osmotic water influx establishes a colloid osmotic pressure gradient over the NM that mediates rapid nuclear swelling. Cells try to restore their original volume by regulatory volume decrease (RVD) which involves the expulsion of small osmolytes and water ^13^. Accordingly, the initial increase of INM-bound ALPS was followed by ALPS dissociation from the INM during the RVD phase (Figure S4d–e). Reversible ALPIN-INM interactions were associated with reversible ER fragmentation (Figure S5b). Irrespective of whether the ALPS emission from the INM was normalized to a fluorescent nuclear envelope marker (LMNB1) or not, similar binding kinetics were obtained (Figure S4g). Because the green emission channel was reserved for concomitant ER imaging, we henceforth used the non-ratiometric readout for quantifying T_INM_.

In addition to intact cells, we also used digitonin permeabilized cells to measure T_INM_ upon nuclear deformation as previously described ^5,8^ (Figure S4c, h, j). In permeabilized cells, Ca^2+^ can be clamped and the colloid osmotic pressure gradient over an intact NM can be experimentally controlled by adjusting the extranuclear colloid concentration with 360 kD polyvinylpyrrolidone (PVP360) in the lysis buffer. PVP360 dilution caused a stepwise increase in ALPIN-INM adsorption (Figure S4h). As expected, the binding of recombinant ALPS1–2-eGFP to osmotically swollen GUVs (Figure S4i) as well as ALPIN-INM interactions in permeabilized cells were independent of Ca^2+^ (Figure S4j). Thus, colloid nuclear swelling increases T_INM_. In line with its larger hydrophobic binding surface, the doublet ALPS motif (ALPS1–2-mKate2 3XNLS) within the nucleus, bound stronger to the osmotically stretched INM (Figure S4e, g **left vs right**). However, this motif was also strongly sequestered by small, curved perinuclear vesicles in the cytoplasm (Figure S4d, **lower panel**), complicating automated image analysis. Interestingly, cytoplasmic ALPS1–2-mKate2 (i.e., without 3XNLS) showed little accumulation on the outer nuclear membrane (ONM) upon osmotic shock (Figure S4f). The curvature sensitivity of ALPS domains is based on their affinity for hydrophobic lipid packing defects ^14^, which also underlies tension sensing ^8^. So, it remains unclear whether its lack of ONM adsorption reflects a lack of ONM tension or sensor sequestration by curved cytoplasmic vesicles away from a tensed ONM. Either way, the nuclear compartmentalization of amphiphilic domains, away from curved cytoplasmic vesicles, is expected to enhance their partitioning into stretched membranes. Indeed, it was previously shown that forcing cPla_2_ into the cytoplasm with a nuclear export sequence, impairs its nuclear shape sensing function ^1^.

### ER fragmentation by osmotic shock and ferroptosis correlates with high T_INM_

Just like cPla_2_, ALPIN revealed a strong correlation of INM-adsorption and ER fragmentation (Figure 1c–f, Supplementary Video 2) upon osmotic shock of intact cells, suggesting that T_INM_ (irrespective of Ca^2+^) drives both ALPIN and cPla_2_ translocation, and is attenuated by a contiguous ER. Irreversible ER fragmentation was typically associated with stable ALPIN translocation, and transient ER fragmentation with reversible ALPIN-INM interactions (Figure S5).

**Figure 2.**
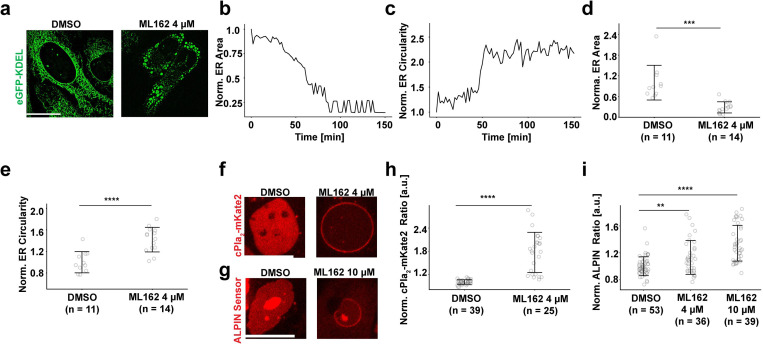
ER fragmentation by osmotic shock and ferroptosis correlates with high T_INM_. **(a)** Representative images of U2OS cells, expressing the ER lumen marker eGFP-KDEL, treated with the glutathione peroxidase 4 (GPX4) inhibitor ML162 (4 μM). **(b)** Quantification of time-dependent ER surface area change upon 4 μM ML162 treatment representative of three different FOVs. **(c)** Quantification of ER circularity upon 4 μM ML162 treatment representative of three different FOVs. **(d)** Comparison of ER surface area between cells treated with DMSO (n = 11) and 4 μM ML162 (n = 14). Error bars, SD. **(e)** Comparison of ER circularity between cells treated with DMSO (n = 11) and 4 μM ML162 (n = 14). Error bars, SD. **(f)** Representative images of cPla_2_-mKate2-INM adsorption in cells treated with DMSO or 4 μM ML162. **(g)** Representative images of ALPIN-INM binding in cells treated with DMSO or 4 μM ML162. **(h)** Comparison of cPla_2_-mKate2-INM binding in cells treated with DMSO (n = 39) or 4 μM ML162 (n = 25). Error bars, SD. **(i)** Quantification and comparison of ALPIN-INM binding in cells incubated with DMSO (n = 53), 4 μM ML162 (n = 36), or 10 μM ML162 (n = 39). Error bars, SD. Scale bars, 20 μm. P values in (d), (e), (h) and (i) are determined by a two-sided Student’s *t*-test assuming unequal variance. For quantification of ER morphology, n represents FOVs each containing ~1–10 cells/nuclei. For measurement of cPla_2_-mKate2- and ALPIN-INM adsorption, n represents number of nuclei.

Cell- and nuclear swelling are hallmarks of necrosis and occur shortly before cells lyse—thus the alternative term *oncosis* (ὄγκος, Greek for bulk/tumor). cPla_2_ accumulation on the INM has been observed during the pre-lytic, *oncotic* phase of lipid peroxide induced necrosis (i.e., ferroptosis) ^15,16^. Yet, these experiments did not discriminate whether cPla_2_ adsorption was caused by changes in Ca^2+^ or T_INM_, nor did they report on ER fragmentation. To clarify this important point, we concomitantly monitored T_INM_ and ER morphology using ALPIN and eGFP-KDEL, respectively. Ferroptosis was triggered by suppressing glutathione peroxidase 4 activity with ML162 ^17 15^. After ~ one hour of ML162 incubation, we observed strong ER fragmentation (Figure 2a–e) along with the recruitment of cPla_2_ and ALPIN to the INM (Figure 2f–i). Lack of concomitant Sytox Orange staining confirmed that all these changes occurred independent of cell lysis (Figure S3b). These data argue for T_INM_ as driver of cPla_2_ mediated nuclear shape sensing during osmotic shock and cell death.

### ER fragmentation escalates T_INM_ during colloid osmotic shock

**Figure 3.**
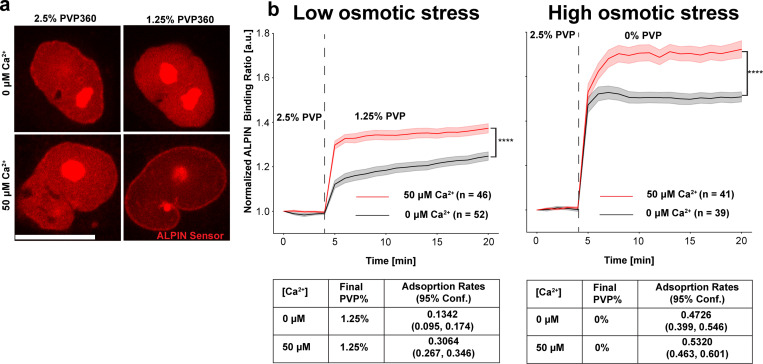
ER fragmentation escalates T_INM_ upon colloid osmotic shock. **(a)** Representative images of ALPIN-binding to NM under colloid osmotic stress (2.5% PVP to 1.25% or 0% PVP) in the presence (0 μM Ca^2+^) or absence (50 μM Ca^2+^) of contiguous ER membrane. **(b)** Left panel, timeseries quantification of ALPIN adsorption to the INM under mild colloid osmotic stress (2.5% PVP to 1.25% PVP) with connected ER membranes (0 μM Ca^2+^, n = 52) or with fragmented ER membranes (50 μM Ca^2+^, n = 46). Transparent color, SEM. Right panel, timeseries quantification of ALPIN binding to NM upon high colloid osmotic stress (2.5% PVP to 0% PVP) with intact ER membranes (0 μM Ca^2+^, n = 39), or with fragmented ER membranes (50 μM Ca^2+^, n = 41). The ALPIN-INM adsorption rates were determined by the Ordinary Least Squares (OLS) of the linear portion of the binding plots. Shaded regions, SEM. Scale bars, 20 μm. P values in (b) are determined by a two-sided Student’s *t*-test assuming unequal variance. For measurement of ALPIN-INM adsorption, n represents the number of nuclei.

The above experiments revealed a striking correlation between ER fragmentation and T_INM_ but not causality. To this end, we experimentally perturbed ER-contiguity in permeabilized cells with Ca^2+^ and used ALPIN to measure T_INM_ upon osmotic shock as a function of ER contiguity (Figure S6a). First, we confirmed that 50 μM Ca^2+^ in the cell permeabilization buffer effectively vesiculated the ER (Figure S6b–d). At moderate colloid osmotic pressure shifts (2.5%→1.25% PVP360, Figure 3), the rate of ALPIN adsorption to the INM suggested a faster rise of T_INM_ in nuclei with a vesiculated (50 μM Ca^2+^) compared to nuclei with a contiguous (no Ca^2+^) ER. But this difference became negligible at higher osmotic differentials. The maximal T_INM_ increased with the osmotic differential and was consistently higher in nuclei with a disrupted ER (Figure 3b, right panel).

### ER fragmentation correlates with stable cPla_2_ translocation in live zebrafish

We previously observed stable cPla_2_ translocation in the wound margin cells of injured zebrafish larvae ^4^. By contrast, transient cPla_2_ translocation typically occurred more distant from the laser injury and was enhanced by latrunculin mediated F-actin depolymerization, suggesting that nuclear deformation and T_INM_ upon osmotic shock are restrained by the actin cytoskeleton ^5^. To probe the correlation of ER-fragmentation with reversible or irreversible cPla_2_ translocation *in vivo*, we injured latrunculin-pretreated zebrafish larvae expressing both cPla_2_-mKate2 and eGFP-KDEL with a pulsed UV-laser as previously described ^4,5^.

**Figure 4.**
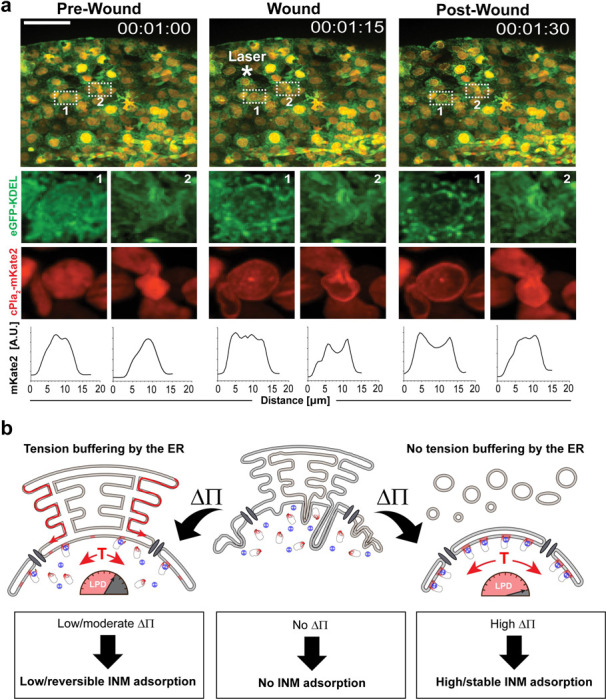
ER fragmentation correlates with stable cPla_2_ translocation in live zebrafish. **(a)** Top, confocal maximum intensity projection of cPla_2_-mKate2 and luminal ER marker eGFP-KDEL in Latrunculin A pretreated zebrafish larvae after wounding under hypoosmotic conditions (π ~ 10 mOsm) at t = 00:01:15. Middle panels, insets show cells within (position 1) and just outside (position 2) the wound region. Scale bar, 50 μm. Bottom panels, profile plots of cPla_2_-mKate2 emission of the depicted nuclei. Constitutive cPla_2_ translocation with ER fragmentation was observed in n = 5 wounding experiments. Reversible cPla_2_ translocation without ER fragmentation was observed in n = 4 wounding experiments. Timestamp, hh:mm:ss. **(b)** Hypothetical scheme. Osmotic shock (DP) must unfold nuclear membrane invaginations (middle panel) before tension (T) can develop. When the ER is contiguous with the nuclear membrane (left panel), a compensatory lipid flow (red dashed line) partially relives T. This amounts to less lipid packing defects (LPD, red shaded regions/gauge) on the INM, and less, or more reversible, hydrophobic cPla_2_-INM interactions, as observed in cultured cells exposed to moderate osmotic stress, in distal wound cells (position 2), or in perivascular macrophages (see Gelashvili et al., 2024) *in vivo*. When the ER is vesiculated (right panel), there is no compensatory lipid flow to attenuate T and lipid packing defects. This amounts to high (and more stable) cPla_2_-INM interactions. The respective cell is turned into a constitutive, inflammatory lipid signaling hub. This is observed, e.g., in wound margin cells (position 1), pre-lytic ferroptotic cells, and post-lytic (i.e., permeabilized) cell corpses.

Upon injury, epithelial cells (large round nuclei) around the laser injury underwent rapid ER fragmentation, which in many but not all cases was associated with stable cPla_2_ translocation (Figure 4a, position 1, Supplementary video 3). That is, ER fragmentation enhances, but is not sufficient for stable cPla_2_ translocation. Other cells, e.g., more distal to the wound, retained an intact ER and, if any, showed transient cPla_2_ INM-interactions (Figure 4a, position 2), akin to those observed in (non-latrunculin treated) perivascular macrophages in our companion paper (Gelashvili et al., 2024). So, ER vesiculation is correlated with stable cPla_2_ activation at zebrafish wound sites *in vivo*. Altogether, our work demonstrates that the ER buffers T_INM_ and nuclear membrane mechanotransduction (Figure 4b).

## Discussion

Here, we use the Ca^2+^ independent T_INM_ biosensor ALPIN to reveal T_INM_ as a central nuclear shape signal. Whereas Ca^2+^ transients may derive from a plethora of mechanical and chemical signaling events without carrying much specificity for the respective trigger, T_INM_ selectively emerges when cells and their nuclei become critically deformed to a degree that threatens cell- and tissue integrity. Unlike Ca^2+^ alone, T_INM_ along with Ca^2+^, specifically reports on critical cell body deformation ^6^. It is thus suited to instruct adaptive responses in cells and tissues, such as cell evasion from physical confinement ^1–3^, rapid immune defense of wounds ^4,5^, and serum perfusion of damaged tissues (Gelashvili et al., 2024).

Although the ER is contiguous with the INM, it does not behave like a regular nuclear membrane fold. Its structural stabilization likely prevents the ER from collapsing into the INM when the latter is stretched. Notwithstanding, the ER controls the amplitude and possibly the reversibility of T_INM_ along with nuclear membrane mechanotransduction, for instance, by enabling a delayed compensatory lipid flow to dissipate T_INM_. In line with previous findings ^11^, ER vesiculation after hypoosmotic shock is partially reversible, and in our cell culture experiments, reversible cPla_2_-INM interactions were typically associated with partially reversible ER fragmentation. Critical osmotic stress, however, abrogates this reversibility, which may explain the correlation of stable ER fragmentation and cPla_2_ translocation in wound margin cells *in vivo*, or pre-lytic ferroptotic cells in culture. The reversibility of ER-fragmentation and T_INM_ is likely multifactorial: Osmotic cell swelling itself is reversible, i.e., in intact cells, it is rapidly counteracted by expulsion of small osmolytes and water during regulatory volume decrease ^13^. Likewise, cytoplasmic Ca^2+^ increase, which modulates both ER fragmentation and cPla_2_-INM adsorption, is usually transient in intact cells. Finally, compensatory lipid flow from an intact ER into the INM likely attenuates T_INM_ with some delay. Here, and in our companion paper (Gelashvili et al., 2024), we found reversible cPla_2_ translocation to the INM always associated with a contiguous ER or partially reversible ER fragmentation.

Our data shows that a contiguous ER does not preclude and instead buffers T_INM_ and nuclear membrane mechanotransduction, likely contributing to their rapid reversibility. Conversely, ER fragmentation (e.g., at wound margins and during necrosis/ferroptosis) turns pre-lytic cells and nucleated cell corpses into stable signaling hubs with continuous inflammatory lipid output. Indeed, stable cPla_2_ activation at the wound margin or in necrotic cells controls the acute wave of neutrophil recruitment within ~ the first hour after injury ^4,5^. The molecular mechanism of ER vesiculation remains largely unknown and is beyond the scope of this study. Our unpublished attempts to disrupt ER morphology by overexpressing or deleting published ER shape factors ^18,19^ never severed the ER from the nucleus as hypoosmotic shock, cell compression, or high Ca^2+^ did. Understanding the mechanism of ER vesiculation may open new therapeutic avenues to attenuate sterile inflammatory responses to necrotic cell death and other severe homeostatic perturbations by restraining nuclear membrane mechanotransduction.

## Methods

### Cell culture and generation of stable cell lines.

U2OS (HTB-96, ATCC) cells were grown in Dulbecco’s Modified Eagle Medium (DMEM) supplemented with 10% fetal bovine serum, 2 mM L-glutamine, and 1% Penicillin-Streptomycin. Lipofectamine 3000 (Thermofisher) was used to transfect U2OS cell lines according to the manufacturer’s instructions. To establish stable transgenic cell lines, cells were co-transfected with SB100X *Sleeping Beauty* transposase and a pSB/MCS/Puro transposon vector encoding the genes of interest under the control of the EF1-α or CMV promoter in 2:1 ratio. 48 hours after transfection, cells were grown in complete growth medium with 1 μg/ml puromycin for 1–2 weeks. mKate2 and eGFP double positive cells were sorted by flow cytometry.

### Lipids.

1,2-Dipalmitoyl-*sn*-glycero-3-phosphocholine (DPPC) and 1,2-dioleoyl-sn-glycero-3-phosphoethanolamine-N-(lissamine rhodamine B sulfonyl) (18:1 Liss Rhod PE) were purchased from Avanti Research.

### Expression vector construction.

For bacterial expression, human ARFGAP1 ALPS1–2-eGFP (human ARFGAP1, amino acids AA192–304) were cloned into a modified pGEX 6P-1 backbone, leading to N-terminal GST fusion with Human Rhinovirus-3C (HRV-3C) protease cleavage sites.

For mammalian expression, rat ARFGAP1 ALPS1-mKate2 (rat ARFGAP1 ALPS1-mKate2, AA196–234) and human ARFGAP1 ALPS1–2-mKate2 (human ARFGAP1 AA192–304) were fused to eGFP-laminB1 or eGFP-Sec61β through P2A peptides on the C-terminus and subsequently cloned into the pSB/CMV/MCS/Puro transposon plasmid as previously described ^5^. Within this plasmid, an expression cassette containing genes of interests is driven by a constitutive CMV promoter, along with another expression cassette that encodes a puromycin selection marker under the control of synthetic RPBSA promoter. Both expression cassettes are flanked by inverted terminal repeats that contain transposase recognition sites and allow transposition into host genome when co-expressed with the hyperactive *Sleeping Beauty* transposase (SB100X). Zebrafish cPla_2_-mKate2 was fused to eGFP-KDEL through a P2A peptide and subsequently cloned into a similar pSB/EF-1 α/MCS/Puro transposon plasmid, in which the genes of interests were under the control of a constitutive EF-1 α promoter.

### Preparation of GUV membranes.

To generate GUV membranes composed of 99.5% DPPC and 0.5% 18:1 Liss Rhod PE, we used electroformation as previously described^8,20^. Briefly, 0.11 μM of lipid mixture dissolved in chloroform was spread on 30 to 60 Ω indium tin oxide (ITO) coverslips (Structure Probe Inc. supplies) and dried under a N2 stream. To completely remove chloroform, the cover slips were exposed to vacuum overnight. ITO coverslips were assembled into an electroformation chamber with 500 mM sucrose solution inside and incubated at 60 °C. The chambers were connected to a function generator (RIGOL DG1022). A sine wave with a frequency of 10.0 Hz and peak-to-peak voltage at 1.41 V was applied for 4–6 h followed by a square wave with a frequency of 4.5 Hz and peak-to-peak voltage at 2.12 V for 30 min. After electroformation, GUVs were extracted from the chamber using a gel loading tip. The chamber was washed two times with 500 mM glucose solutions to collect residual GUVs. GUVs were used for experiments within 5 days after preparation.

### Recombinant protein expression and purification.

GST fusions of C-terminally tagged ARFGAP1-ALPS1–2-eGFP were expressed and purified as previously described ^21^. The pGEX-6P-1 expression vector harboring tagged ARFGAP1-ALPS was transformed into Rosetta 2 pLySs cells (Novagen). Expression of ARFGAP1-ALPS was induced by 0.2 mM IPTG at 37 °C under vigorous shaking (250 rpm) for 3 h in LB medium supplemented with Ampicillin (100 μg/mL) at OD600 = 0.8. After expression, bacteria were resuspended, sonicated, and centrifuged. Supernatants were incubated with Glutathione Sepharose 4B gel beads (Cytiva) overnight at 4 °C. The beads were washed three times in a buffer containing 25 mM HEPES (pH =7.4) and 200 mM KCl afterward. Beads with GST-tagged ARFGAP1-ALPS were incubated with HRV-3C protease (PreScission Protease, Cytiva) in a buffer containing 25 mM HEPES (pH =7.4) and 200 mM KCl overnight at 4 °C to elute ARFGAP1-ALPS1–2. Eluates were concentrated with Amicon Ultra-2 10K centrifugal filter devices. The total protein concentration was determined using the Pierce BCA Protein Assay Kit. Protein purity was determined by SDS-PAGE and Coomassie blue staining. Bands were quantified in Fiji. Aliquots were snap frozen in liquid nitrogen and stored at −80 °C.

### GUV equilibrium binding experiments.

The binding experiments were performed as previously described ^8^. Concentrated GUVs were transferred to a hypoosmotic binding buffer containing 0 or 20 μM Ca^2+^ (as specified in the table below) and ALPS1–2-eGFP domains at varying concentrations (ranging from 0 to 500 nM) in bovine serum albumin (BSA)-coated glass-bottom dishes (20 mg/ml BSA solution was added 1–2 hours before the experiment to reduce GUV membrane ruptures during incubation). GUVs were left in the binding solution for two hours until protein recruitment approached an equilibrium. Afterward, GUV membrane and protein fluorescence images were simultaneously acquired using a 40× objective and GUV membrane fluorescence was quantified and normalized as described in “Image acquisition and computational analysis for cell culture experiments” section.

The apparent dissociation constant ${K_{d}}^{\prime }$, maximum binding intensity $B_{\max}$, and the apparent Hill coefficient H were determined by nonlinear least-square analysis of bound-protein fluorescence $\left(B_{\text{bound}}\right)$ versus the total domain concentration ([Domain]) using Langmuir adsorption isotherm with Hill expansion:

$$B_{bound}=B_{max}\left(\frac{{\left[Domain\right]}^{H}}{{\left[Domain\right]}^{H}+{K_{\prime d}}^{H}}\right)$$

**Table:** 

${\text{Δ M}}_{\text{osm}}$	Salt Concentration	Sucrose Concentration	Free [Ca^2+^]
240 mOsm	113.5 mM KCl, 1 mM MgCl_2_, 5 mM EGTA/K^+^, 10 mM HEPES/K^+^, addition of KOH to maintain pH ~ 7.4	0 mM	0 μM
240 mOsm	106 mM KCl, 1 mM MgCl_2_, 5 mM EGTA/K^+^, 10 mM HEPES/K^+^, 5 mM CaCl_2_, addition of KOH to maintain pH ~ 7.4	0 mM	20 μM

### Hypoosmotic treatment experiments.

96 well glass bottom dishes (Cellvis) were coated with 0.1% gelatin in PBS w/o Ca^2+^ and Mg^2+^ for at least 15 min at 37°C before washing 3X with PBS. U2OS cells stably expressing fluorescent markers were then seeded in 96 well dishes (~3 × 10^4^ cells/well for 96 well) 24 h before the start of the imaging experiments in the specified cell culture solutions (see above). Before the experiment, the cell culture medium was exchanged to Leibovitz’s L-15 medium (ThermoFischer). Under isosmotic conditions, U2OS cells were incubated in L-15 stock culture medium to obtain the final osmotic pressure of 341 mOsm. To induce different levels of hypoosmotic shock, the cell imaging medium was diluted by addition of double-distilled water supplemented with 1.26 mM CaCl_2_ according to the table below. For timelapse imaging experiments, cells were first imaged under isosmotic conditions for 4 – 5 min before applying the hypoosmotic shock. Cells were imaged for 20 min. To evaluate plasma membrane integrity during osmotic swelling, an additional experiment was conducted where 50 nM Sytox Orange (Molecular Probes) was included in the culture medium and cells were imaged with/without the application of osmotic swelling.

To compare the effects of hypoosmotic shock on stretching of nuclear envelopes and ER structures in a large population of cells, a single time lapse frame consisting of both nuclear and ER membranes was captured 5 min after hypoosmotic shock treatment.

**Table:** 

Starting volume of medium	Volume of dilution medium	Final Osmotic Shock (Δπ)
80 μL	320 μL	270 mOsm
40 μL	360 μL	303 mOsm

### Nuclear compression.

To compress the cell nucleus and study the effects on ER structure and T_INM_, 30 mL of 2% Agarose (Fisher Scientific) in Leibovitz’s L-15 medium was prepared, brought to boil, and transferred to a 100 mm petri dish (Fisher Scientific) to solidify. A 1 cm-radius circle agarose pad was cut out and gently deposited on U2OS cells stably expressing cPla_2-_mKate-2P2A-eGFP-KDEL pre-equilibrated in L-15 medium. Specifically, cells with the agarose pad on top were first imaged for 5 mins before carefully adding a 15 g weight on the pad while continuously imaging for another 55 min. To make sure no plasma membrane leakage took place during mechanical perturbation, an additional experiment was performed where 50 nM Sytox Orange was included in the medium and cells with/without mechanical compression were imaged and compared for Sytox Orange staining of nucleus. As a positive control for the Sytox Orange staining, plasma membranes were ruptured by strong mechanical compression (weight = 20 g).

To compare the effects of mechanical compression on ER network morphology and T_INM_ in a large population of cells, a single time point image of cells with/without weight was captured 30 mins after applying the weight on top of cells.

### Cell permeabilization experiments.

The permeabilization protocol was modified from a previously established protocol^8^. Specifically, the base medium (“BM”) was prepared for permeabilization experiments under Ca^2+^-free condition: 123 mM KCl, 12 mM NaCl, 1 mM KH_2_PO_4_, 1.94 mM MgCl_2_, 10 mM MOPS and 10 mM EGTA/Na^+^ at pH 7.3–7.4. For permeabilization in the presence of Ca^2+^, CaCl_2_ was added to a modified BM (i.e., all ingredients remain the same as in BM, except for decreasing EGTA/Na^+^ to 1 mM). Unless otherwise noted, cell membranes were permeabilized in BM/modified BM supplemented with 12.5 μg/mL Digitonin (Cayman Chemical) and 2.5% PVP360 (Millipore Sigma) for > 60 mins before imaging to make sure that the nuclear envelope is unstretched at the start of imaging. Decreasing PVP360 concentration was used to induce and control T_INM_. To test the role of ALPS1-mKate2 3XNLS (ALPIN) as a T_INM_ sensor and measure the temporal dynamics of ALPIN sensor binding to the nuclear envelope upon increasing colloid osmotic shock, permeabilized cells were first incubated and imaged in BM medium supplemented with 2.5% PVP360 for 5 min. Permeabilized cells were then washed 2x with PBS followed by incubating and imaging in BM supplemented with PVP360 concentrations ranging from 0% to 2.5% for 10 min. To rule out the possibility of ALPS1-mKate2 3XNLS binding to stretched NM being affected by Ca^2+^, permeabilized cells were first imaged in modified BM supplemented with 2.5% PVP360 and 10 μM Ca^2+^, or 50 μM Ca^2+^ for 5 mins before diluting PVP360 concentration to 0% while maintaining the same [Ca^2+^] and continuing imaging for 15 mins.

To measure the effect of increasing [Ca^2+^] on ER network morphology in permeabilized cells, cells were first imaged in L-15 stock medium for 5 min, followed by incubation and imaging in BM supplemented with 12.5 μg/mL Digitonin and 2.5% PVP360 (Ca^2+^-free) or modified BM supplemented with 12.5 μg/mL Digitonin, 2.5% PVP360 and additional CaCl_2_ (50 μM Ca^2+^) for 20 min. To compare the effects of [Ca^2+^] concentrations and T_INM_ on ER structure in a large population of cells, permeabilized cells were incubated for one hour under the following conditions: (a) BM supplemented with 2.5% PVP360 (Ca^2+^-free and not causing colloid osmotic stretch). (b) BM alone (Ca^2+^-free and causing osmotic stretch). (c) Modified BM supplemented with 2.5% PVP360 and additional CaCl_2_ (50 μM Ca^2+^ and not causing colloid osmotic stretch). (d) Modified-BM supplemented with additional CaCl_2_ (50 μM Ca^2+^ and causing osmotic stretch). A single time point image of ER structure under four different conditions was captured after incubation.

To measure T_INM_ in permeabilized cells with or without contiguous ER, permeabilized cells were imaged in BM supplemented with 2.5% PVP (= ER network remains intact) or modified BM supplemented with 2.5% PVP and 50 μM Ca^2+^ (= ER network is fragmented and disconnected from NM) for 5 min before diluting the PVP360 concentration to 1.25% or 0% at constant [Ca^2+^]. The imaging was continued for 15 min.

### Ferroptosis induction.

To study ER fragmentation and T_INM_ during ferroptosis, cells were imaged in L-15 medium supplemented with DMSO or 4 μM glutathione peroxidase 4 inhibitor ML162 (Selleckchem). Imaging was performed over 2.5 h. 50 nM Sytox Orange was added to the medium to test whether the ER and T_INM_ changes occur before or after cell lysis. To quantitatively compare ER structure and T_INM_ between normal and ferroptotic cells in a large population, single time frames of cells incubated in L-15 supplemented with 4 or 10 μM ML162, or DMSO were captured at 1.5 hours after incubation.

### Zebrafish Husbandry.

Adult wt and transgenic reporter casper ^22^ zebrafish were maintained as described ^23^ and subjected to experiments according to institutional animal healthcare guidelines with the approval of the Institutional Animal Care and Use Committee (IACUC). The adult Tg(*hsp70i*:cPla_2_-mKate2-P2A-eGFP-KDEL) fish were reared in 2.8 polycarbonate tanks at animal density 10 fish L^−1^. The anesthesia for the offspring was all conducted with 0.2 mg ml^−1^ 3-amino benzoic acid ethyl ester (Sigma, MS-222, E10521), (pH=7.0), buffered in 0.5 mg ml^−1^ anhydrous sodium phosphate dibasic (Fisher, BP332–500). The embryos were staged by dpf (days-post fertilization). Sex was indeterminate at 2.5–4 dpf and all in vivo experiments in the manuscript were conducted at these embryonic stages. The animal embryos were collected from natural spawning and raised in standard hypoosmotic E3 containing 0.1%(w/v) methylene blue (Sigma-Aldrich, M9140) for first 24hrs followed by E3 medium (5 mM NaCl(Sigma-Aldrich, S7653), 0.17 mM KCl (Sigma-Aldrich, P9333), 0.33 mM CaCl_2_ (Sigma-Aldrich, C5670), 0.33 mM MgSO_4_ (Sigma-Aldrich, M7506)) in 100 mm petri dishes(Fisher Scientific, FB0875713) in complete darkness.

### Transgenesis, plasmid construction and *in vitro* transcription.

Fertilized casper zebrafish embryos were collected and injected at the one-cell stage into the cytoplasm with a Nanoject II^™^ microinjector (Drummond Scientific, Broomball, PA). Plasmids were assembled using Gateway multisite cloning kit using LR Clonase^™^ (Invitrogen; C12537–023) and multicloning sites between the gateway att sites flanking cassette of p5E (*hsp70i*), pME(cPla_2_-mKate2) and p3E(P2A-eGFP-KDEL) plasmids that were recombined into Tol2kit Destination pDESTcrybb1 for generating Tg(*hsp70i*:cPla_2_-mKate2-P2A-eGFP-KDEL). The middle entry pME vector was assembled from cytosolic phospholipase A2 (cPla_2_, Ensembl: ENSDARG00000024546), open reading frame amplified by PCR and ligated into pDONOR221 containing pME gateway compatible att sites and subsequently fused in-frame to mKate2 (Evrogen) as a C-terminus fluorescent tag with a 15 amino-acid GS-enriched linker. The p3E entry vector bears a self-cleaving peptide in the N_TD_ of eGFP that is followed by ER localization signal KDEL and SV40 pA. For transgenesis, Tol2kit transposase mRNA was transcribed from NotI linearized pCS2FA-transposase plasmid with mMESSAGE mMACHINE SP6 reverse transcription kit (Thermo Scientific, AM1340). The final transgenesis construct was injected into one cell stage zebrafish embryos (~2.7 nL/embryo) at a concentration of 25 ng μL^−1^ along with 25 ng μL^−1^ Tol2kit transposase mRNA. Among the injected embryos, fluorescence-positive siblings in the eye were selected and raised in husbandry and backcrossed at sexual maturity to Casper fish and their progeny used for larvae experiments.

### Intravital confocal microscopy and laser wounding of zebrafish larvae.

For laser wounding experiments, two-day post fertilization (dpf) Tg(*hsp70i*:cPla_2_-mKate2-P2A-eGFP-KDEL) were heat shocked at 37 °C for 2 hours to induce transgene expression. The fluorescent embryos were sorted out for experiments 12–18 hours after heat treatment using a coaxial dissection stereomicroscope (MVX10, Olympus) equipped with a mercury lamp (LM200B1-A, Prior Scientific) and set dichroic mirrors (MVX-RFA; 540/35 U-MGFPHQ/XL, 625/55 U-MRFPHQ/XL). The selected embryos were anesthetized in hypoosmotic E3 medium ^23^ containing 0.2 mg ml^−1^ 3-amino benzoic acid ethyl ester (Sigma, MS-222, E10521), (pH=7.0), buffered in 0.5 mg ml^−1^ anhydrous sodium phosphate dibasic (Fisher, BP332–500). Using ~200 μL 1% low-melting (LM) agarose dissolved in E3, the anesthetized 3 dpf embryos were embedded on their right side in a 60 mm plastic petri dish (Corning, 351007). The solidified LM-agarose was covered with ~2–3 mL standard E3 medium, or preincubated for 30 min in E3 supplemented with ethanol as vehicle (1% EtOH in E3), or latrunculin A (2.5 μM, Sigma-Aldrich, L5163) to create a submerging environment for the 25x objective lens (N.A.=1.1 ꚙ/0–0.17 WD= 2 μm Water Dipping, Nikon) in upright microscope. The samples were excited with 488 and 561 nm diode lasers (Andor Revolution XD). Excitation intensity/exposure was set to 35%/80 ms (488 nm) and 30%/80 ms (561 nm). ER and cPla_2_-mKate2 fluorescence was acquired using a Andor iXon3 897 EMCCD camera mounted on a Nikon Eclipse FN1 microscope (25x objective lens; N.A.=1.1, ꚙ/0–0.17 WD= 2 μm, Nikon) equipped with Yokogawa CSU-X1 spinning disk unit (pinhole = 50 μm), an optovar (1.25x magnification) and NIS imaging software (NIS Elements, 3.22.14). Emission was acquired through band-pass filters for green (525/40, Semrock, FF02–525/40–25) or red (617/73, Semrock, FF02–617/73–25) fluorescence emission spectra. Confocal stacks were collected at 1.5 μm z step size and repeated in no-delay intervals per position for up to 5 min. The wounds were induced at t~1 min with several successive laser pulses targeted at periphery of the epithelium using a microscope-mounted 435 nm Micropoint laser (Andor).

### Image processing for zebrafish experiments.

For contrast enhancement, the 4D image stacks of Tg(*hsp70i*: cPla_2_-mKate2-P2A-eGFP-KDEL) were subjected to blind-3D deconvolution (Landweber) using Nikon NIS elements (5.21.03, Build 1489). The deconvolved or MP image stacks are denoised using denoise.ai tool for improving signal-to-noise in Nikon NIS elements (5.21.03). Profile plots of cPla_2_-mKate2 emission at the indicated time points were generated using in Fiji(v1.54J) “line” and “Plot Profile” tool functions.

### Image acquisition and computational analysis for cell culture experiments.

The dynamics of cPla_2_ and ALPIN bindings and ER structures were measured using a Nikon Eclipse Ti inverted spinning-disk confocal microscope with a Plan Apo 100×/1.45 oil objective or a Plan Apo 40×/0.95 air objective and equipped with a Yokogawa CSU-X1 Spinning Disk unit, an ANDOR iXon ULTRA 897BV EMCCD camera, 488 nm, and 561 nm solid-state laser lines (Andor Revolution) and NIS-Elements Software (4.13.04). mKate2 emission was excited at 561 nm and collected through a 620 bandpass emission filter (590 to 650 nm, Chroma Technology., 49005; 8800v2 Quad Set). eGFP fluorescence was excited at 488 nm and collected through a 525 band-pass emission filter (515 to 555 nm, Chroma Technology, 49002;8800v2 Quad Set). Identical microscope settings were used throughout imaging. Experiments involving GUVs, and permeabilized cells took place at room temperature. Live imaging of cultured cells was performed at 37°C. Nikon’s Perfect Focus System (PFS) was enabled throughout the imaging session. For timelapse imaging experiment, Z-stacks (1.4–2 μm steps over 30 μm) were acquired each minute. For single time point acquisition, the single Z-stacks were acquired at the end of the experiment. For high resolution imaging of the ER network, we used a structured illumination microscope (SIM, 27.5 μm G5).

Images were acquired using Elyra 7 Zeiss Microscope with a 63x Objective (Plan-Apochromat 63x N.A.=1.4 ꚙ/0.17 WD=0.19 mm Oil, 420782–9900-000 Zeiss) oil immersion lens. Emission was excited at 488 nm with beam splitters (SBS LP 560) and sampled through a 495–550 nm band pass filters (LBF 405/488/561/642, ) and each emission for red and green were collected on two separate sCMOS camera (Excelitas technology, pco.edge 4.2 M) and aligned during preprocessing in Zeiss software (Zen Black 3.0 SR FP2, v16.0.20.306). A single Z-stack (0.8 μm steps over 30 μm) was acquired at the end of the experiment. All images were taken in the identical microscope settings. The SIM reconstruction was conducted using the Zeiss Software (Zen Black 3.0 SR FP2). All 3D segmentation and analysis were performed with the Anaconda distribution of Python (Python ≥ 3.7). Specifically, customized Python scripts were developed using the Numpy^24^, Scipy^25^, Scikit image^26^, Allen Cell Structure Segmenter^27^, trackpy ^28,29^and Napari libraries^30^.

To quantify binding of ALPS 1–2 eGFP to GUV membranes, a custom, semiautomatic Python script was developed. First, a line was manually drawn by the user from the center of GUV to the edge to crop a selected GUV from the whole image stack. Next, intensity normalization and a three-dimensional Gaussian blur (3-pixel radius) were applied to the cropped GUV stack to remove background noise. A marker-controlled watershed algorithm was used on each slice of the cropped stack to segment the GUV. The slice with largest segmented GUV area was defined as the middle section. Based on the watershed segmentation result, a 3-pixel-wide contour of the GUV was drawn onto the GUV middle section. Lastly, the rim-binding intensity was calculated as the median of all intensities along the contour. To account for fluorescence variabilities across multiple purification batches, raw rim-binding of each domain was normalized by the fluorescence signal of a 1 μM protein solution from same purification batch. Detailed codes are available at: https://github.com/joeshen123/GUV-Protein-Binding-Analysis-Program.git.

A similar, fully autonomous Python pipeline was developed to segment the nucleus in 3D and measure NM bindings of cPla_2_-mKate2 and ALPIN sensor. Firstly, intensity normalization, 3D gaussian blur (1-pixel radius) and rolling-ball algorithms were applied to the images to remove uneven background noise. Secondly, a masked-object thresholding (this algorithm was employed to separate foreground object from background of the 3D image stack through automatically determined threshold and generate a marker-image that distinguish individual nucleus) followed by a marker-controlled watershed algorithms were used to segment the NM within the 3D image stack. Since the movement of cell nuclei was limited (less than 50 μm) during the duration of the experiment, a standard particle tracking algorithm was utilized to track individual nucleus over time. To measure the NM protein binding ratio, the slice with largest segmented nuclear area for was selected as representative, middle section for each nucleus. Based on the watershed segmentation result of the middle section, a 3-pixel-wide contour (“membrane contour”) was drawn onto the NM and another 3-pixel-wide contour (“background contour”) was drawn to mark the inside of nucleus (nucleoplasm). The binding was calculated by dividing the medians of all intensities along the “membrane contour” by the median of all intensities along the “background contour” in the channel where protein fluorescence was measured. For normalization, the protein binding of each nucleus over time was divided by the protein binding at the start of the imaging (t=0 min). One caveat from this type of imaging analysis is that an increase of binding ratio could be due to a decrease of intensity in nucleoplasm instead of an increase of protein binding to the NM. To account for this issue, an alternative ratiometric binding was calculated between median intensity along the “membrane contour” of the 561 channel (protein of interests is tagged with mKate2) and median intensity along the “membrane contour” of the 488 channel (LMNB1 nuclear marker is tagged with eGFP, the intensity of eGFP-LMNB1 remains constant throughout the imaging session). Detailed codes are available at: https://github.com/joeshen123/Nuclear-Membrane-Binding-Analysis.git.

To quantify ER structure, a python-based automatic image analysis pipeline was developed. Specifically, the segmentation algorithm of choice was slightly different depending on the specific tag used to label the ER. With luminal labeling (e.g., eGFP-KDEL), ER was segmented by a marker-controlled watershed algorithm the same way as NM segmentation. If ER membranes were labelled (e.g., Sec61β), the image was processed by a filament filter algorithm that segments dense filamentous and network-like structures in the cell. ER surface areas were extracted from the segmentation masks and circularity was calculated according to the following equation: $Circularity=4\pi \times \frac{\mathrm{Surface Area}}{{\mathrm{Perimeter}}^{2}}$ (0 for highly non-circular object and 1 for perfect circle). For each time point, median values of all surface areas and circularities in one field of view (FOV) were calculated. Because the ER membranes of neighboring cells were in close proximity, it was technically difficult to segment the ER for individual cells. Instead, the ER was quantified per FOV. To account for the variation of ER between different FOVs, the fold change of median surface area and circularity over time were normalized to the value at the start of the experiment (t=0 min). Detailed codes are available at: https://github.com/joeshen123/ER-Structure-Analysis.git.

### Statistics.

For fitted coefficients of ARFGAP1 ALPS1–2 equilibrium binding, the error bars denote the 95% confidence interval (CI). For timelapse comparison of ER circularity, cPla_2_- and ALPIN-NM binding, the error bars denote the standard error of mean (SEM). Otherwise, error bars represent the standard deviation (SD). Detailed sample sizes are listed in the figure legends. A two-tailed Students *t* test assuming unequal variances from Python’s Scipy *ttest_ind* function was used to determine *P* values (*P≤0.05, **P≤0.01, ***P≤0.001, ****P≤0.0001, ns P>0.05). ALPIN-NM binding rates were estimated via ordinary least squares (OLS) on the linear part of the binding curves from Python’s Statsmodel package ^31^. Graphical plots were generated using Python’s matplotlib^32^ and seaborn libraries^33^. Helical-wheel representation of ALPS1 motif structure in Figure S4 was created using Python’s matplotlib library as well. Since the ER circularity/area was quantified per field of view (FOV) while binding ratio of cPla_2_-mKate2 and ALPIN sensor was calculated per cell/nucleus, in the scatter correlation plots, different cells within the same FOV were given the same ER circularity/area value.

## Supplementary Material

Supplement 1
