# Supplementary material for: Buffering of nuclear membrane tension and mechanotransduction by the endoplasmic reticulum revealed by quantitative ALPIN imaging
Source: Res Sq. 2024 Dec 9:rs.3.rs-5530637. Preprint. [Version 1] doi: 10.21203/rs.3.rs-5530637/v1 (PMC11661296; doi:10.21203/rs.3.rs-5530637/v1)
Supplement: Supplement 1 [file NIHPPRS5530637V1-supplement-1.pdf]

Figure S1

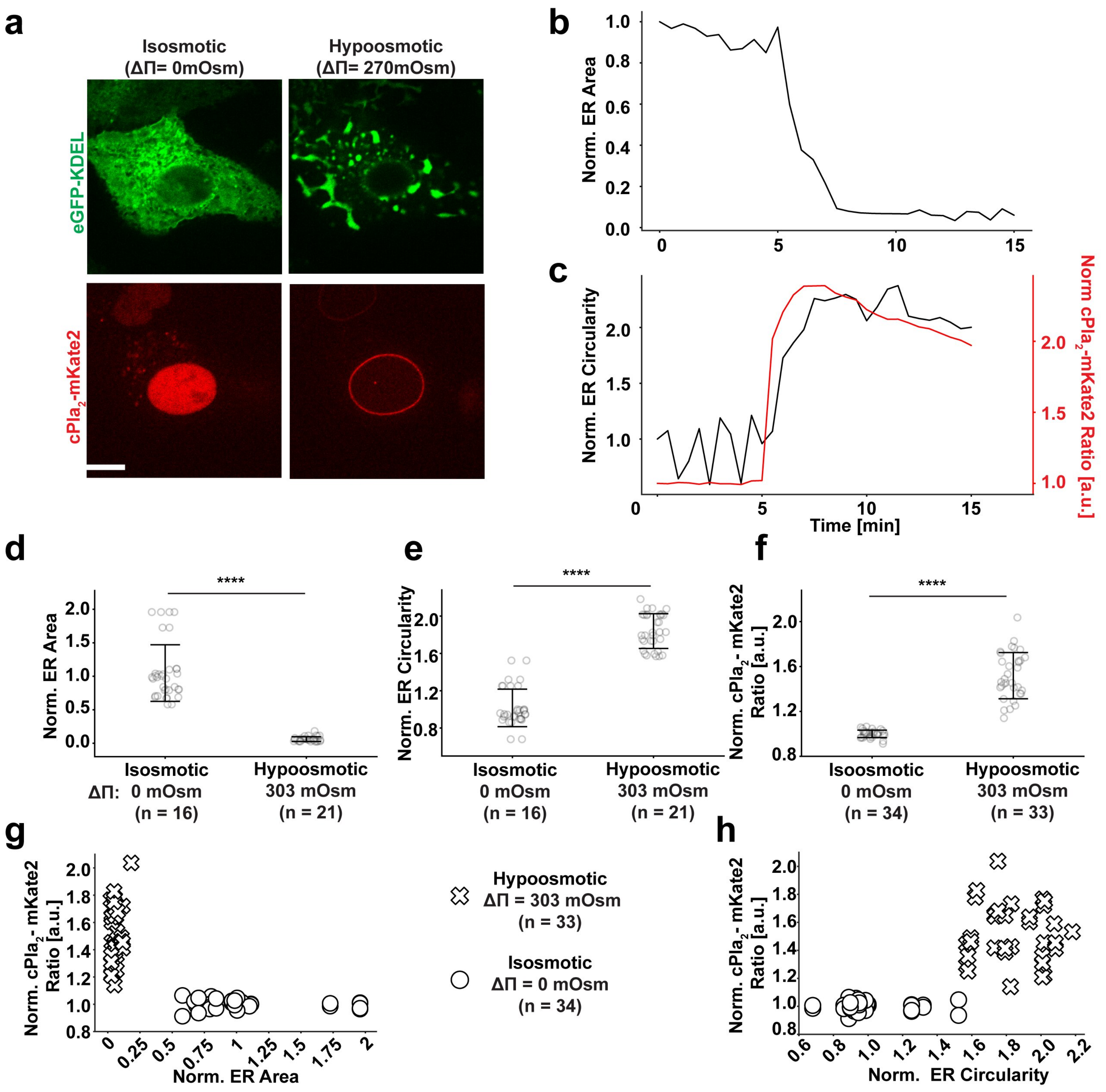

a
Isosmotic (ΔΠ= 0mOsm)
Hypoosmotic (ΔΠ= 270mOsm)
eGFP-KDEL
cPla2-mKate2
b
Norm. ER Area
c
Norm. ER Circularity
Norm cPla2-mKate2 Ratio [a.u.]
Time [min]
d
Norm. ER Area
****
Isosmotic
ΔΠ: 0 mOsm
(n = 16)
Hypoosmotic
303 mOsm
(n = 21)
e
Norm. ER Circularity
****
Isosmotic
0 mOsm
(n = 16)
Hypoosmotic
303 mOsm
(n = 21)
f
Norm. cPla2- mKate2 Ratio [a.u.]
****
Isosmotic
0 mOsm
(n = 34)
Hypoosmotic
303 mOsm
(n = 33)
g
Norm. cPla2- mKate2 Ratio [a.u.]
Norm. ER Area
Hypoosmotic
ΔΠ = 303 mOsm
(n = 33)
Isosmotic
ΔΠ = 0 mOsm
(n = 34)
h
Norm. cPla2- mKate2 Ratio [a.u.]
Norm. ER Circularity

Figure S1. Extended data related to Figure 1. (a) Representative images of U2OS cells expressing $cPla_2$-mKate2 and the ER lumen marker eGFP-KDEL, exposed to isosmotic ($\Delta\pi$ = 0 mOsm) and hypoosmotic ($\Delta\pi$ = 270 mOsm) medium. **(b)** Plot of ER surface area vs. time after hypoosmotic shock treatment ($\Delta\pi$ = 270 mOsm). **(c)** Plot of ER circularity (black line) and $cPla_2$-mKate2-INM adsorption (red line) vs. time after hypoosmotic shock ($\Delta\pi$ = 270 mOsm). (b, c) Both plots are representative of n = 58 hypoosmotically shocked cells. **(d)** Quantification of ER network surface area of cells treated with isosmotic (n = 16) and hypoosmotic (n = 21) solution. Error bars denote the SD. **(e)** Comparison of ER circularity between cells treated with isosmotic ($\Delta\pi$ = 0 mOsm, n = 16) or hypoosmotic ($\Delta\pi$ = 270 mOsm, n = 21) medium. Error bars denote the SD. **(f)** $cPla_2$-mKate2-INM binding in cells treated with isosmotic ($\Delta\pi$ = 0 mOsm, n = 34) or hypoosmotic ($\Delta\pi$ = 270 mOsm, n = 33) medium. Error bars represent the SD. **(g)** Scatter plot showing the correlation between $cPla_2$-mKate2-INM adsorption and ER vesiculation (as measured by decrease of ER surface area) in cells exposed to hyposmotic ($\Delta\pi$ = 303 mOsm, n = 34) or isosmotic medium ($\Delta\pi$ = 0 mOsm, n = 33). Note, the correlation plot includes data plotted in (d), (e) and (f). **(h)** Scatter plot showing the correlation between $cPla_2$-mKate2-INM binding and ER fragmentation (as measured by an increase of ER network circularity) in cells exposed to hypoosmotic ($\Delta\pi$ = 303 mOsm, n = 34) or isosmotic solution ($\Delta\pi$ = 0 mOsm, n = 33). Note, the correlation plot is generated from raw data depicted in (d), (e) and (f). Scale bars, 20 µm. P values in (d), (e) and (f) are determined by a two-sided Student's *t*-test assuming unequal variance. For quantification of ER structure, n represents different FOVs with ~ 1-10 cells each. For measurement of $cPla_2$-mKate2 NM binding, n denotes the number of nuclei. For the scatter plots in (g) and (h), n represents number of nuclei. Note, different nuclei in the same FOV have the same ER circularity/area value (i.e., protein adsorption is assessed per nucleus, whereas ER circularity is assessed per FOV, see Methods).

Figure S2

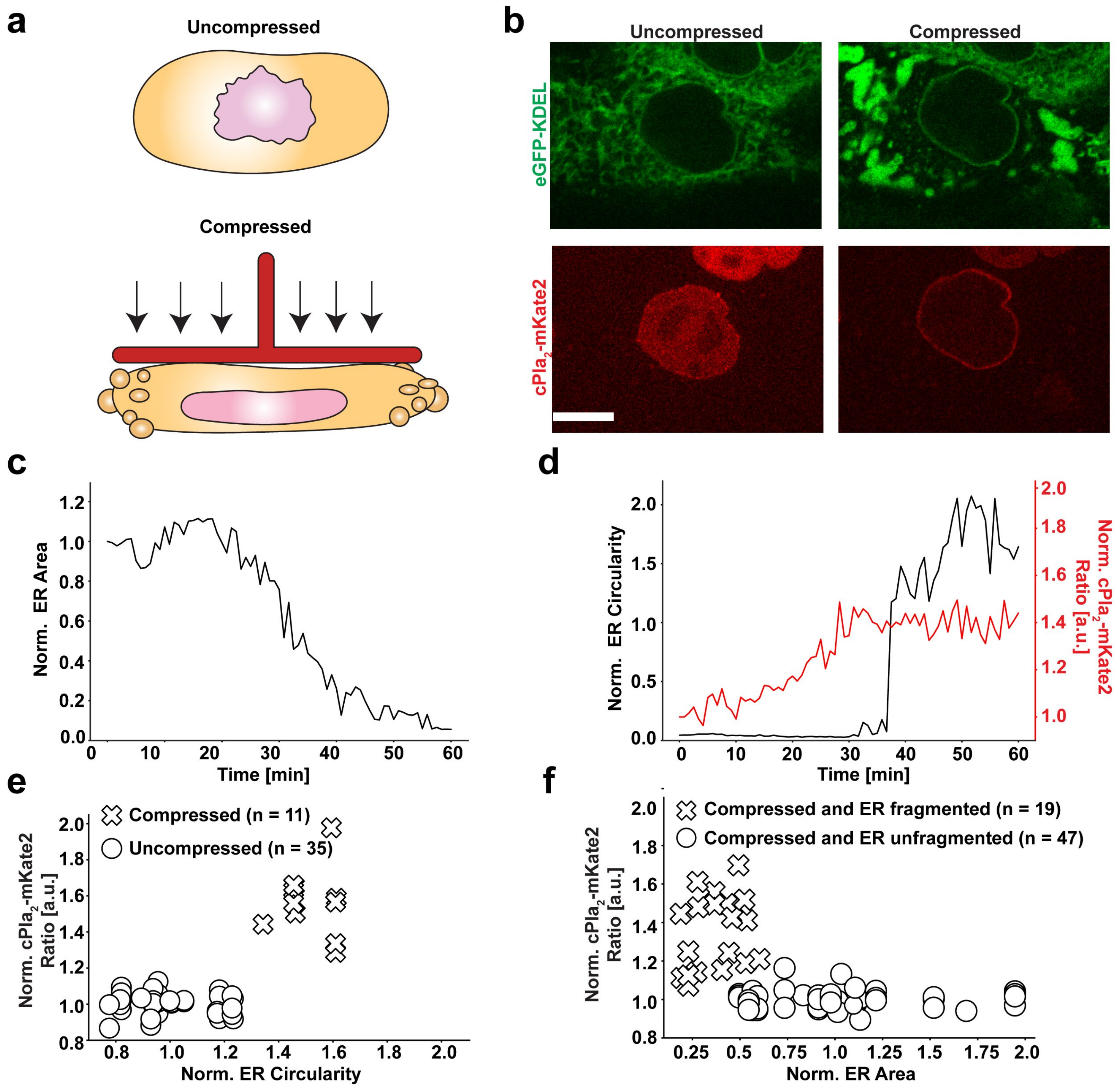

a
Uncompressed
Compressed
b
Uncompressed
Compressed
eGFP-KDEL
cPla2-mKate2
c
Norm. ER Area
Time [min]
d
Norm. ER Circularity
Norm. cPla2-mKate2 Ratio [a.u.]
Time [min]
e
Compressed (n = 11)
Uncompressed (n = 35)
Norm. cPla2-mKate2 Ratio [a.u.]
Norm. ER Circularity
f
Compressed and ER fragmented (n = 19)
Compressed and ER unfragmented (n = 47)
Norm. cPla2-mKate2 Ratio [a.u.]
Norm. ER Area

Figure S2. Extended data related to Figure 1. **(a)** Cartoon depiction of cell compression experiment. **(b)** Representative images of U2OS cells expressing $cPla_2$-mKate2 and the ER lumen marker eGFP-KDEL upon compression with a 15 g weight. **(c)** Plot of ER surface area vs. time during mechanical compression. **(d)** Plot of ER circularity (black line) and $cPla_2$-mKate2-INM binding (red line) during mechanical compression. (c-d) Plots are representative of n = 13 compressed cells. **(e)** Scatter plot of $cPla_2$-mKate2-INM binding vs. ER vesiculation (as measured by an increase of ER circularity) in compressed (n = 11) or uncompressed cells (n = 35). **(f)** Scatter plot of $cPla_2$-mKate2-INM binding vs. ER vesiculation (as measured by a decrease of ER network area) in compressed cells with fragmented ER (n = 19) vs. compressed cells with intact ER (n = 47). Scale bars, 20 µm. For the scatter plots in (e) and (f), n denotes the number of nuclei. Different nuclei in the same FOV are assigned the same ER circularity/area value (see Methods).

Figure S3

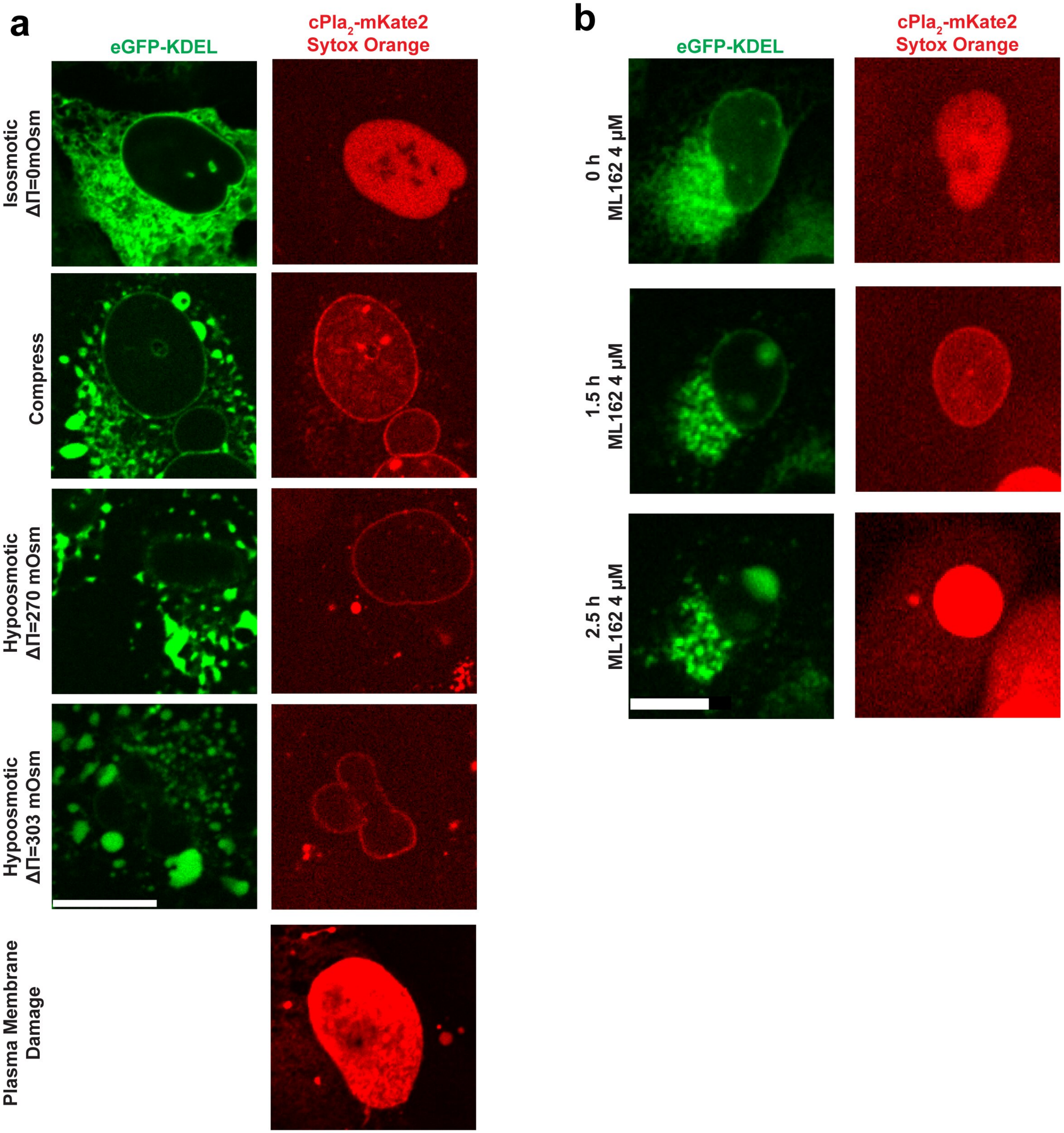
a
eGFP-KDEL
cPla2-mKate2
Sytox Orange
Isosmotic
ΔΠ=0mOsm
Compress
Hypoosmotic
ΔΠ=270 mOsm
Hypoosmotic
ΔΠ=303 mOsm
Plasma Membrane
Damage
b
eGFP-KDEL
cPla2-mKate2
Sytox Orange
0 h
ML162 4 μM
1.5 h
ML162 4 μM
2.5 h
ML162 4 μM

Figure S3. Extended data related to Figure 1 & 2. (a) Representative images of U2OS cells expressing cPla$_2$-mKate2 and the ER lumen marker eGFP-KDEL upon osmotic swelling or cell squeezing in the presence of the lysis marker Sytox Orange. First row, under isosmotic conditions, nuclear fluorescence represents nucleoplasmic cPla$_2$-mKate2; second row, under mechanical compression the ER vesiculates and cPla$_2$-INM adsorbs to the INM, but Sytox Orange does not stain nuclei; third/fourth row, under mild or strong hypoosmotic shock ($\Delta\pi$ = 270/303 mOsm) nuclei remain Sytox orange negative; fifth row, positive control with plasma membrane ruptured by strong mechanical compression (weight = 20 g). **(b)** Representative images of U2OS cells expressing cPla$_2$-mKate2 and the ER lumen marker eGFP-KDEL upon 4 µM ML162 treatment. Scale bars, 20 µm.

Figure S4

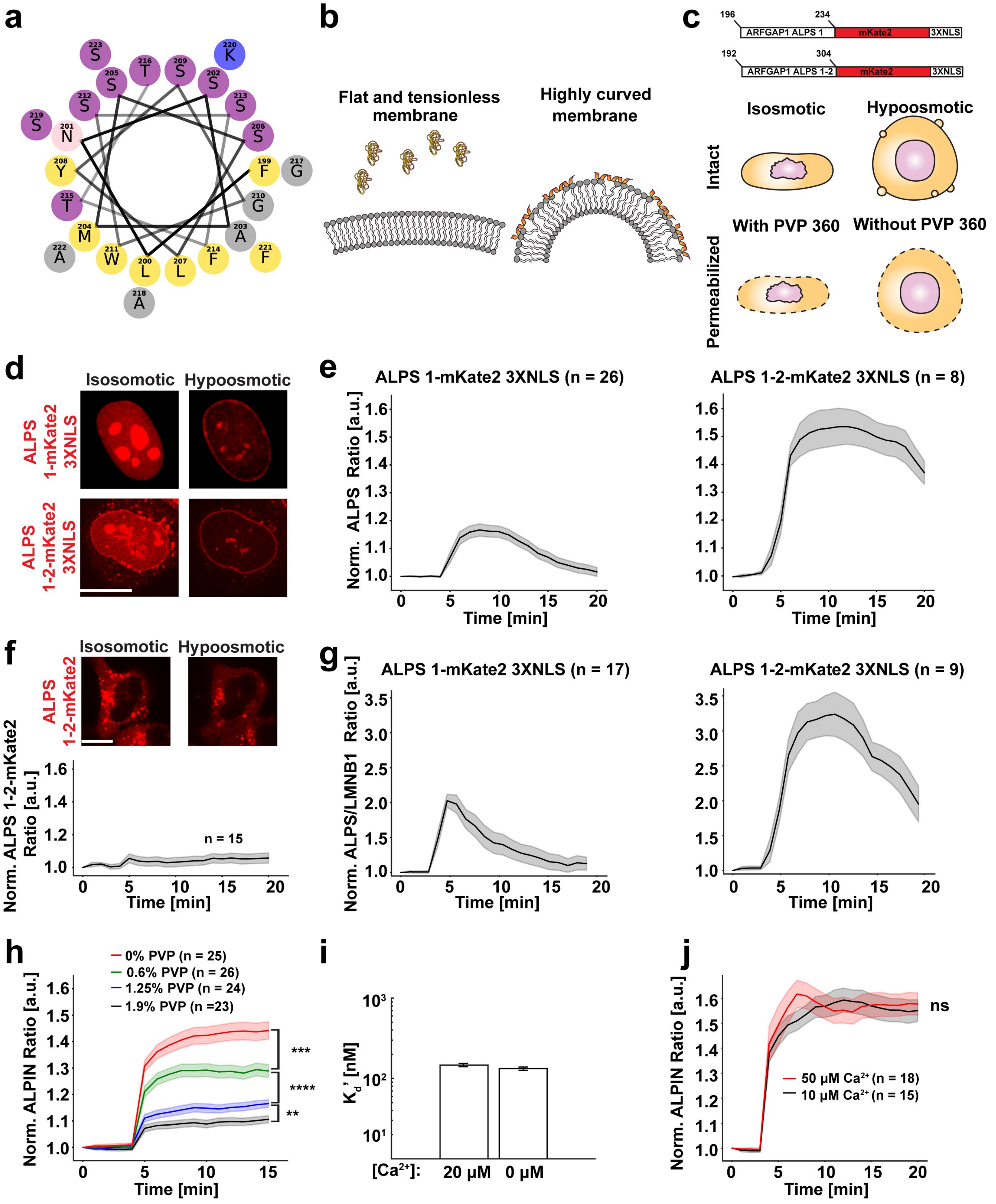
a
b
Flat and tensionless membrane
Highly curved membrane
c
196
234
ARFGAP1 ALPS 1
mKate2
3XNLS
192
304
ARFGAP1 ALPS 1-2
Isosmotic
Hypoosmotic
Intact
With PVP 360
Without PVP 360
Permeabilized
d
Isosomotic
Hypoosmotic
ALPS 1-mKate2 3XNLS
ALPS 1-2-mKate2 3XNLS
e
ALPS 1-mKate2 3XNLS (n = 26)
ALPS 1-2-mKate2 3XNLS (n = 8)
Norm. ALPS Ratio [a.u.]
Time [min]
f
ALPS 1-2-mKate2
Norm. ALPS 1-2-mKate2 Ratio [a.u.]
n = 15
g
ALPS 1-mKate2 3XNLS (n = 17)
ALPS 1-2-mKate2 3XNLS (n = 9)
Norm. ALPS/LMNB1 Ratio [a.u.]
h
0% PVP (n = 25)
0.6% PVP (n = 26)
1.25% PVP (n = 24)
1.9% PVP (n =23)
Norm. ALPIN Ratio [a.u.]
***
****
**
i
K_d' [nM]
[Ca²⁺]:
20 μM
0 μM
j
ns
50 μM Ca²⁺ (n = 18)
10 μM Ca²⁺ (n = 15)

Figure S4. Extended data related to Figure 1-3. (a) Cartoon representation of the ARFGAP1 ALPS1 motif. Yellow, hydrophobic; purple, serine and threonine; grey, glycine and alanine; blue, basic residues. **(b)** Hypothetical cartoon scheme of ALPS interaction with flat or curved membranes. **(c)** Top panel, scheme of the ALPS-based T_{INM} biosensors used in this study. Bottom panel, cartoon representation of experimental strategies for osmotic deformation of the nucleus **(d)** Representative images of ALPS1-mKate2 3XNLS (= ALPIN) (top) and ALPS1-2-mKate2 3XNLS (bottom) -INM interactions before and after hypoosmotic shock ($\Delta\pi$ = 270 mOsm). **(e)** Sensor adsorption (left, ALPIN, n = 26; right, ALPS 1-2-mKate2 3XNLS, n = 8) to the INM after hypoosmotic shock ($\Delta\pi$ = 270 mOsm). **(f)** Top, representative images of ALPS1-2-mKate2 (without NLS) binding to the ONM before and after hypoosmotic shock ($\Delta\pi$ = 270 mOsm). Bottom, timeseries quantification of INM adsorption (n = 15). **(g)** Timeseries quantification of ALPIN (left, n = 17) and ALPS 1-2-mKate2 3XNLS (right, n = 9) binding to the nuclear rim upon hypoosmotic shock ($\Delta\pi$ = 270 mOsm), normalized by eGFP-LMNB1 fluorescence. **(h)** Timeseries quantification of ALPIN adsorption to the INM after dilution of [PVP360] from 2.5% to 1.9% (n = 23), 1.25% (n = 24), 0.6% (n = 26) or 0% (n = 25) under Ca^{2+}-free conditions. Shaded region, SEM. **(i)** Bar graph of the apparent dissociation constants (K_d') of ALPS 1-2-eGFP -GUV interactions at 0 or 20 µM Ca^{2+} upon hypoosmotic shock ($\Delta\pi$ = 240 mOsm). The apparent dissociation constants were determined from n > 450 GUV measurements (across different ALPS concentrations) by fitting a Langmuir/Hill equation. Scale bar, 95% confidence interval. **(j)** Timeseries quantification of ALPIN-INM interactions at 50 (n = 18) or 10 µM Ca^{2+} (n = 15) after diluting [PVP360] from 2.5% to 0% (= maximal/extreme colloid osmotic shock). Scale bars, 20 µm. P values in (h) and (j) were determined by a two-sided Student's t-test assuming unequal variance. For measurements of ALPIN or ALPS 1-2-mKate2 3XNLS-INM interactions, n denotes the number of measured nuclei.

Figure S5

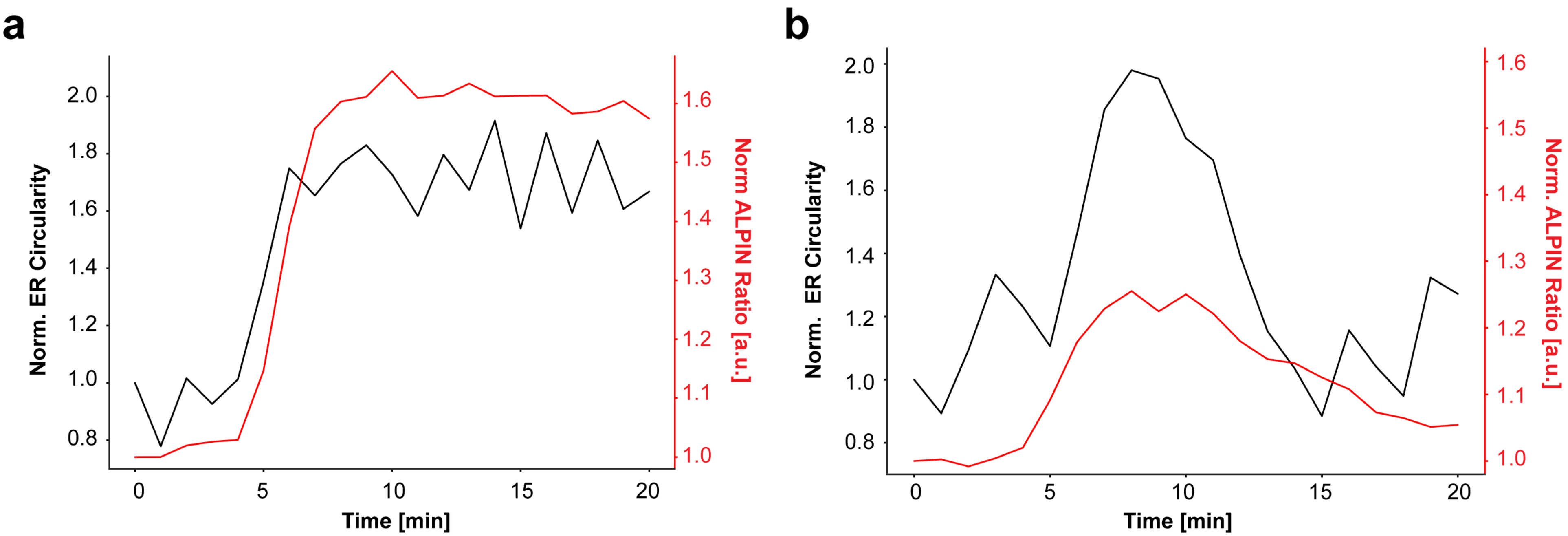

a
Norm. ER Circularity
Norm ALPIN Ratio [a.u.]
Time [min]
b
Norm. ER Circularity
Norm. ALPIN Ratio [a.u.]
Time [min]

Figure S5. Extended data related to Figure 1. Representative timeseries quantification of ALPIN-INM interaction (red line) and ER network circularity (black line) in U2OS cells exposed to hypoosmotic shock ($\Delta\pi$ = 270 mOsm). **(a)** Cells with ER fragmentation show persistent ALPIN-INM interactions. The plot is representative of n = 42 cells. **(b)** Transient ER fragmentation correlates with transient ALPIN-INM interactions. This plot is representative of n = 43 cells.

Figure S6

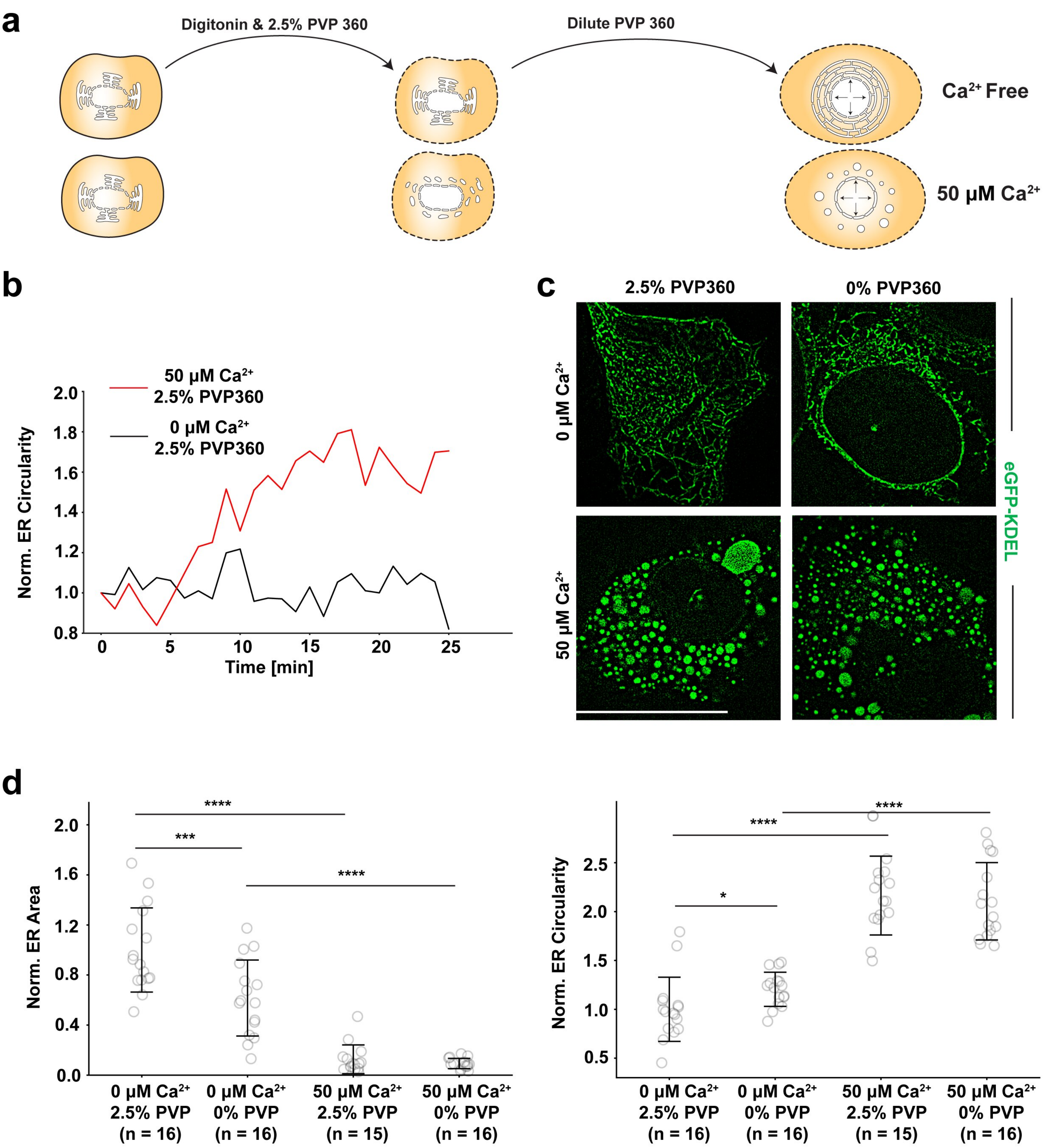
a
Digitonin & 2.5% PVP 360
Dilute PVP 360
Ca2+ Free
50 μM Ca2+
b
50 μM Ca2+
2.5% PVP360
0 μM Ca2+
2.5% PVP360
Norm. ER Circularity
2.0
1.8
1.6
1.4
1.2
1.0
0.8
0
5
10
15
20
25
Time [min]
c
2.5% PVP360
0% PVP360
0 μM Ca2+
50 μM Ca2+
eGFP-KDEL
d
Norm. ER Area
2.0
1.6
1.2
0.8
0.4
0.0
****
***
****
0 μM Ca2+
2.5% PVP
(n = 16)
0 μM Ca2+
0% PVP
(n = 16)
50 μM Ca2+
2.5% PVP
(n = 15)
50 μM Ca2+
0% PVP
(n = 16)
Norm. ER Circularity
2.5
2.0
1.5
1.0
0.5
****
****
*
0 μM Ca2+
2.5% PVP
(n = 16)
0 μM Ca2+
0% PVP
(n = 16)
50 μM Ca2+
2.5% PVP
(n = 15)
50 μM Ca2+
0% PVP
(n = 16)

Figure S6. Extended data related to Figure 3. (a) Cartoon scheme explaining the permeabilized cell experiments presented in Figure 3 **(b)** Representative timeseries quantification of ER circularity upon digitonin permeabilization under calcium-free conditions or with 50 µM Ca^{2+}. 2.5% PVP was added to keep nuclei osmotically balanced by compensating for the loss of cytoplasmic proteins during lysis. **(c)** Representative images of U2OS cells expressing the ER lumen marker eGFP-KDEL exposed to different concentrations of Ca^{2+} and PVP360. **(d)** Quantification of ER area (left panel) and circularity (right panel) after digitonin-permeabilization as a function of [Ca^{2+}] and [PVP360]. Error bars, SD. Scale bars, 20 µm. P values in (d) are determined by a two-sided Student's t-test assuming unequal variance. For quantification of ER structures, n represents the number of FOVs with ~1-10 cells each.

Supplementary Videos

Supplementary Video 1. U2OS cell expressing cPla$_2$-mKate2 and eGFP-KDEL under hypoosmotic treatment ($\Delta\pi$ = 270 mOsm)**.** cPla$_2$-mKate2-INM adsorption correlates with ER vesiculation after hypoosmotic shock. Red, cPla$_2$-mKate2. Green, eGFP-KDEL (ER-marker). Scale bar, 20 µm.

Supplementary Video 2. U2OS cell expressing ALPIN and eGFP-Sec 61β under hypoosmotic treatment ($\Delta\pi$ = 270 mOsm)**.** ALPIN-INM adsorption correlates with ER vesiculation after hypoosmotic shock. Red, ALPIN biosensor. Green, eGFP-Sec 61β (ER-marker). Scale bar, 20 µm.

Supplementary Video 3. Confocal maximum intensity projection of cPla$_2$-mKate2 (red) and luminal ER marker eGFP-KDEL (green) in a latrunculin A pretreated zebrafish larva after wounding under hypoosmotic conditions (π ~ 10 mOsm) at t = 00:01:15. Middle panels, insets show cells within (rectangle 1) and just outside (rectangle 2) the wound region. Timestamp, hh:mm:ss. Scale bar, 50 µm.

Supplementary Files

This is a list of supplementary files associated with this preprint. Click to download.

- SupplementaryVideo3compressed.avi
- SupplementaryVideo2.avi
- SupplementaryVideo1.avi
